# Analgesic treatment for refractory cancer pain caused by gastric cancer bone metastasis: A case report and literature review

**DOI:** 10.1097/MD.0000000000038851

**Published:** 2024-07-12

**Authors:** Dan Wang, Ting Jiang, Lingli Zheng, Chaomin Liu, Xiaomei Fan, Yan Li, Jing Li

**Affiliations:** a Clinical Medical College, Chengdu Medical College, Chengdu City, Sichuan Province, China; b Department of Pharmacy, The First Affiliated Hospital of Chengdu Medical College, Chengdu City, Sichuan Province, China; c Department of Oncology, The First Affiliated Hospital of Chengdu Medical College, Chengdu City, Sichuan Province, China.

**Keywords:** adverse reactions, cancer, case analysis, opioid medications, pain management, serotonin syndrome

## Abstract

**Rationale:**

Patients with bone metastasis-associated cancer pain often experience a complex mix of pain types. Consequently, the use of multimodal combination therapy is essential. While monitoring for common adverse reactions in pain treatment, it is also crucial to be vigilant for the rare but serious serotonin syndrome.

**Patient concerns:**

A 53-year-old female with metastatic gastric cancer was hospitalized due to severe, uncontrolled thoracic and cervical pain. During the titration of her cancer pain medication, she developed serotonin syndrome.

**Diagnoses:**

He was diagnosed with refractory cancer pain and serotonin syndrome.

**Interventions:**

The complete process of cancer pain medication in a patient with gastric cancer and bone metastasis was analyzed, with a primary focus on the selection of analgesic medications, adjustment of opioid dosages, and prevention and treatment of medication-associated adverse reactions.

**Outcomes:**

The patient’s cancer pain was well controlled, with the prompt management of adverse reactions. Furthermore, by adjusting the medication regimen, intolerable adverse reactions were prevented.

**Lessons:**

In clinical settings, personalized analgesic regimens must be developed for patients with cancer pain to enhance patient compliance with medication, prevent the occurrence of severe adverse reactions, and improve the overall quality of life of patients with cancer. Healthcare professionals should pay increased attention to ADRs associated with opioid medications, whereas pharmacists should assist them in promptly identifying ADRs.

## 1. Introduction

The skeletal system is the third most common site for cancer metastasis, with the vertebrae (69%) being the most common site, followed by the pelvis (41%), long bones (25%), and the skull (14%).^[1]^ Skeletal-related events such as pain, mobility impairment, hypercalcemia, pathological fractures, spinal cord compression, and bone marrow infiltration can decrease the quality of life of patients and increase the risk of complications such as depression and anxiety. Researchers have reported that approximately 75% of patients with cancer experience cancer-induced bone pain (CIBP).^[2]^ CIBP is similar to inflammatory and neuropathic pain. The pathophysiological mechanism of CIBP is complex and multifactorial, involving tumor cells, bone cells, an inflammatory microenvironment, and neuronal tissues. However, the key characteristics that distinguish CIBP from these pain types remain unelucidated, making it difficult to control CIBP. Cancer pain is one of the most common complications of cancer and its treatment, with some gap between the diagnosis and treatment of cancer pain and the health needs of patients.^[3]^ In a study^[4]^ published in 2022, researchers investigated cancer pain incidence in 533 patients with primary malignant tumors and its management by 197 physicians. They reported that the incidence of cancer pain was 45.4%. Furthermore, among the patients who reported pain in the past 24 hours, only 74.4% were taking analgesics. The most common reason for patients not taking analgesics was that the pain was tolerable. On the other hand, the reason for physicians not administering opioid analgesics to patients was owing to concerns about adverse reactions (43.7%) and addiction (43.1%). The treatment goal should be to achieve the lowest possible pain level and improve the quality of life of patients.^[5]^ Therefore, multimodal approaches such as surgical intervention, radiotherapy, and drug therapy are recommended to manage CIBP, with drug therapy being an important treatment modality. Opioids are the gold standard for treating moderate-to-severe cancer pain. When combined with additional adjuvant therapies, sufficient pain alleviation can be achieved in different cancer pain syndromes. At present, although various clinical practice guidelines recommend using various analgesics, they lack personalized recommendations.^[6]^ Therefore, in this study, we provided valuable insights for the safe clinical application of opioid analgesics via the pharmaceutical monitoring of the complete therapeutic process for a patient with gastric cancer and bone metastasis-associated pain. For this, we demonstrated the entire process of analgesic treatment for a patient with refractory cancer pain owing to gastric cancer bone metastasis, providing some reference for individualized analgesic treatment.

## 2. Case description

A 53-year-old woman with metastatic gastric cancer was admitted to the hospital with uncontrolled thoracic back and neck pain. Medical history: More than 4 months before admission, the patient experienced multiple pain in the neck, chest, and back without any evident cause, accompanied by abdominal distension and other discomforts. Gastroscopy revealed a “gastric ulcer type new organism” and poorly differentiated adenocarcinoma. Immunohistochemical staining revealed the following: MSH2 (+), MSH6 (+), PMS2 (+), MLH1 (+), KI-67 (65%), ERBB2 (−), and PD-L (−). In addition, PET-CT revealed a slight thickening of the local gastric wall at the corner of the stomach, with increased glucose metabolism; this was consistent with the indication of a primary tumor. Furthermore, multiple lymph node enlargements were observed in the left neck, chest, and abdomen, as well as multiple bone lesions with abnormally increased glucose metabolism. Accordingly, the patient was diagnosed with gastric antral poorly differentiated adenocarcinoma with retroperitoneal, cervical lymph node, and multiple bone metastases (cT3N3M1, IV). Four cycles of the FLOT chemotherapy regimen were performed. The regimen included continuous intravenous infusion of docetaxel 50 mg/m^2^ ivgtt d1, oxaliplatin 85 mg/m^2^ ivgtt d1, tetrahydrofolate 200 mg/m^2^ ivgtt d1, and 5-FU 2600 mg/m^2^ for 24 hours; q2w; and zoledronic acid 4 mg q28d to prevent bone-related events. Her home pain regimen comprised hydroxycodone hydrochloride sustained-release tablets 30 mg orally every 12 hours and morphine instant-release 10 mg every 12 hours as needed for acute pain. Physical examination: Body temperature: 36.5°C, pulse rate: 80 beats/min, breathing rate: 17 breaths/min, blood pressure: 118/84 mm Hg, and height: 159 cm. The patient exhibited poor mental health, with a weight loss of approximately 5 kg after illness. No other special circumstances were noted. Auxiliary examination: No significant anomalies were noted in blood routine, liver function, kidney function, coagulation function, and blood lipids. Personal history: No history of smoking, drinking, allergies, or special circumstances.

## 3. Treatment adjustments

### 3.1. First treatment adjustments

During the clinic visit, the patient mentioned that her pain score was consistently 7 to 9 and that she had been using the maximum prescribed morphine dose of 15 mg/mouth every 12 hours. Furthermore, she complained of severe pain with numerical rating scale (NRS) pain scores of 7 to 9 on a 0 to 10 scale, DN4 score of 4 on a 0 to 10 scale, and ID Pain score of 4 on a − 1 to 5 scale. In addition, she mentioned that the frequency of explosive pain was 5 to 6 times in the past 24 hours. Her psychological condition indicated severe anxiety.

The nature of the patient pain was considered mixed, including both nociceptive and neuropathic pain. The patient felt that her current pain regimen was “not really touching the pain.” Therefore, an IV sufentanil patient-controlled analgesia (PCA) pump was placed (background amount: 5 μg/h, PCA amount [amount to treat burst pain]:10 μg (10%–20% of the total amount of opioids in the first 24 hours), and lock time: 10 to 15 minutes, limit amount: 1 h = 5 times PCA amount + background amount) along with oral celecoxib 200 mg/day and oral doxepin 25 mg/day. Thereafter, the patient reported slight pain relief, with an average NRS pain score of 7 and breakthrough pain 4 times. Based on the NRS score on the first day, the second-day background dose of sufentanil was adjusted to 10 μg/h, with a PCA amount of 15 μg. On the second day, the patient average pain score was 5 points, with breakthrough pain 4 times. Therefore, the third-day background dose of sufentanil was adjusted to 15 μg/h, with a PCA amount of 20 μg. However, during the third day of medication, the patient developed general convulsions, chills, increased heart rate, shortness of breath, and blood pressure as high as 185/110 mm Hg. The patient has no history of hypertension, and her blood pressure was normal both outside of the hospital and during the initial admission test. On the fourth day of using sufentanil, her systolic blood pressure increased to 185 mm Hg. Because liver and kidney functions were normal during hospitalization, renal parenchymal and renovascular hypertension was ruled out. Therefore, drug-induced hypertension was suspected. The patient current medications were adjusted, and the Naranjo evaluation scale was utilized to evaluate the correlation of adverse reactions. Sufentanil may have caused adverse reactions. Therefore, sufentanil was discontinued, and diazepam 5 mg was immediately administered. However, the patient symptoms were not significantly relieved. After continuing diazepam 10 mg administration, the symptoms were slightly relieved; however, the patient still experienced convulsions and increased blood pressure. Therefore, nitroglycerin 20 mg was continuously pumped intravenously, naloxone 2 mg (5% GS 500 mL) was slowly dripped, and doxepin hydrochloride tablets were discontinued. As a result, the patient symptoms were significantly relieved.

### 3.2. Second treatment adjustments

Considering the patient medication history and adverse reaction symptoms, we believe that the patient developed drug-induced serotonin syndrome. Based on the Naranjo score (7 points), serotonin syndrome may have been owing to sufentanil; however, the synergistic effect of doxepin could not be ruled out. Therefore, we adjusted the analgesic treatment plan as follows: dexmedetomidine was added for combined analgesia. The background doses of sufentanil and dexmedetomidine were both 5 μg/h, with a PCA amount of 15 μg. Celecoxib was adjusted to 0.2 g twice daily, and gabapentin 0.3 g qd was added. After adjusting the analgesic treatment plan, the patient pain was significantly relieved. On the third day of titration, the pain score was 1 to 2 points. The final analgesic plan was as follows: sufentanil 10 µg/h, PCA 20 µg/h, dexmedetomidine 6 µg/h, celecoxib 200 mg twice daily, and gabapentin capsules 0.3 g thrice daily. The patient developed constipation during the titration period. Nevertheless, after symptomatic treatment with lactulose and glycerin enema, no intolerable constipation-related adverse reactions occurred. Table 1 summarizes the entire titration status. During the titration period, the patient also underwent radiotherapy for bone metastases. After the patient condition stabilized, we adjusted the analgesic plan by discontinuing dexmedetomidine and decreasing the dose of sufentanil by 10% to 25% daily. Finally, the stable dose of sufentanil was converted into oral oxycodone extended-release tablets 90 mg every 12 hours, for long-term maintenance treatment. The patient pain was well controlled during follow-up outside the hospital.

**Table 1:** Medication regimen and titration situation.

Time	Method	NRS score	Anxiety state	Adverse reactions	Management of adverse reactions
First day of titration	sufentanil 5 μg/h; PCA amount 10 μg; celecoxib 200 mg/d; doxepin 25 mg/d; Burst pain 5 times	Average NRS score 7 points; burst pain 8 points.	Severe	Constipation	Lactulose
Second day of titration	sufentanil 10 μg/h; PCA amount 15 μg; Burst pain 4 times	Average NRS score 5 points; burst pain 7 points.	Moderate	Constipation	Lactulose
Third day of titration	sufentanil 15 μg/h; PCA amount 20 μg; celecoxib 200 mg/d doxepin 25 mg/d; Burst pain 4 times	Average NRS score 3 points; burst pain 6 points.	Moderate	Increased constipation	Lactulose + Glycerine Enema
Third day of titration	diazepam 5 mg; diazepam10 mg; Nitroglycerin 20 mg; naloxone 2 mg	drμg-induced serotonin syndrome.			
Adjustment of the titration protocol on the first day	sufentanil 5 μg/h; PCA amount 10 μg; dexmedetomidine 5 μg/h; Celecoxib 200 mg bid Gabapentin 0.3 g qd Burst pain 4 times	Average NRS score 5 points; burst pain 7 points.	Moderate	Reduced constipation	Lactulose
Adjustment of the titration protocol on the second day	sufentanil 10 μg/h; PCA amount 20 μg; dexmedetomidine 6 μg/h; Celecoxib 200 mg bid Gabapentin 0.3 g bid Burst pain 2 times	Average NRS score 1–2 points; burst pain 3 points.	Mild	Constipation	Lactulose
Adjustment of the titration protocol on the third day	sufentanil 10 μg/h; PCA amount 20 μg; dexmedetomidine,6 μg/h; Celecoxib 200mg bid Gabapentin 0.3 g tid Burst pain 0 times	Average NRS score 1–2 points.	None	Constipation	Lactulose + Glycerine Enema

## 4. Discussion

### 4.1. Analysis of the rationality of the treatment plans

A comprehensive pain assessment on admission confirmed that the patient pain was caused by bone metastases. Upon tumor metastasis to the bone, the patient risk of fracture, hypercalcemia, and pain increases.^[7]^ In addition to the general mechanisms underlying nociceptive pain, bone metastasis cancer pain is also characterized by special pathophysiological mechanisms, including abnormal bone metabolism, changes in the microenvironment of surrounding tissues, activation of nociceptors, compression of nerves, and damage caused by tumor growth.^[8]^ Therefore, bone metastasis-associated cancer pain often manifests as a mixed pain type, encompassing characteristics of nociceptive, inflammatory, and neuropathic pain, requiring combined analgesic treatment with drugs with different mechanisms.^[8]^

During bone metastasis, tumor, endothelial, and inflammatory cells release an abundant amount of inflammatory substances, sensitizing or directly activating primary afferent neurons. Nonsteroidal anti-inflammatory drugs (NSAIDs) inhibit cyclooxygenase (COX), thereby preventing the conversion of arachidonic acid into prostaglandins and thromboxane; furthermore, they exert antipyretic, analgesic, and anti-inflammatory effects.^[9–11]^ Celecoxib is an NSAID that selectively inhibits COX-2, preventing inflammatory prostaglandin production and achieving antipyretic, analgesic, and anti-inflammatory effects.^[12]^ Preclinical investigations have suggested promising prospects for COX inhibitors in preserving cancer immune function, possibly providing better options for decreasing cancer recurrence and proliferation.^[13]^ Additionally, celecoxib can reverse immune suppression and counteract the proangiogenic effects of opioid medications in mice.^[13]^ Clinical studies have revealed that the combination of diclofenac or celecoxib with opioid drugs can significantly control bone metastasis-associated cancer pain and that celecoxib can alleviate adverse reactions associated with increasing opioid dosage.^[10,14,15]^ Therefore, our application of celecoxib as an adjuvant therapy for the initial analgesic treatment of bone metastasis-associated cancer pain is reasonable and has sufficient evidence.

The patient pain exhibited features of neuropathic pain. Compared with pain induced by injury stimuli, that induced by nerve damage causes greater damage to the physical and mental health of patients. Neuropathic pain is caused by direct damage to the nervous system, with nerve infiltration or crushing by tumors. Once the peripheral nerves are damaged, pain fibers become extremely sensitive. As a result, pain signals are amplified in the spinal cord, and even a slight touch can result in abnormal pain. All guidelines recommend tricyclic antidepressants and anticonvulsants as first-line treatments for neuropathic pain. Anticonvulsants are primarily used for tearing, discharge-like, and burning pain caused by nerve damage. On the other hand, tricyclic antidepressants are primarily used for numbness and burning pain caused by central or peripheral nerve damage. These medications can also improve mood and enhance sleep quality.^[16–18]^ However, the guideline recommendations for antidepressants and anticonvulsants are primarily based on data on non-cancer neuropathic pain, with the absence of good-quality clinical trials in cancer-associated neuropathic pain.^[19]^ Despite relatively limited evidence, gabapentin, pregabalin, amitriptyline, and duloxetine are used to alleviate cancer-associated neuropathic pain^.[20,21]^ However, the selection of such drugs requires individualization, which not only depends on age, drug interactions, and comorbidities but also the coexistence of multiple symptoms in patients with cancer. Therefore, accurately selecting nerve pain drugs to treat more than 1 symptom can avoid multidrug treatment regimens.^[19]^ Considering the patient pain characteristics and their anxious state, we added gabapentin and doxepin to their treatment plan. The analgesic mechanism of gabapentin in neuropathic pain has been hypothesized as follows: antagonism of the α2δ subunit of voltage-dependent calcium channels at the presynaptic sites. The effects of gabapentin and pregabalin are well established in post-herpetic neuralgia, painful diabetic neuropathy, spinal cord injury pain, and neuropathic cancer pain. Furthermore, gabapentin has no known drug-drug interactions and is well tolerated.^[22–25]^ Therefore, our application of gabapentin as an adjuvant therapy for the initial analgesic treatment of neuropathic pain is reasonable and has sufficient evidence.

### 4.2. Analysis of adverse reactions

The patient developed serotonin syndrome as an adverse reaction during the titration period. Serotonin syndrome is a rare, unpredictable, and potentially fatal reason for fever. The incidence of this condition among 15 million patients in the USA who were taking serotonergic agents was approximately 0.07% to 0.19%. However, the actual incidence of serotonin syndrome remains unknown because it is under-recognized and under-reported^.[26]^ Serotonin syndrome occurs because of the overstimulation of serotonin receptors in the central and peripheral nervous systems by serotonergic drugs. Severe serotonin syndrome generally occurs only when 2 or more serotonergic drugs are simultaneously used.^[27]^ Different types of serotonin receptors are distributed on different types of brain neurons. 5-HT1A receptor, 5-HT2A receptor, and 5-HT2C receptor are some important receptors. Previous studies^[28–31]^ have suggested that the mechanism underlying serotonin syndrome development may be primarily associated with the 5-HT2A receptor. 5-HT2A receptors are present in the cerebral cortex, hypothalamus, gastrointestinal tract, blood vessels, and bronchial smooth muscle, resulting in the diverse symptoms of serotonin syndrome. Furthermore, studies have revealed that serotonin syndrome occurrence may be associated with the polymorphisms of the T102C site of the 5-HT2A receptor and CYP2D6.^[28–31]^ Moreover, serotonin syndrome occurrence is closely associated with the patient medication, disease, and genetic status, with a low incidence; however, when combined with medication for patient cancer pain, its incidence considerably increases. Therefore, for patients with cancer pain and high-risk factors, we should closely monitor serotonin syndrome occurrence and take active prevention measures for patients with suspicious symptoms.

In 2016, the Food and Drug Administration released drug safety information regarding the association between opioid drugs and serotonin syndrome and stated that opioid drugs may cause serotonin toxicity, particularly when used in combination with other serotonin drugs. All opioid drugs are not serotonin reuptake inhibitors; however, they may act on serotonin releasers via the following mechanisms: by slightly inhibiting serotonin reuptake, and by increasing synaptic serotonin release, opioid drugs do not directly stimulate serotonin neurons to increase serotonin release; however, they inhibit γ-GABA and neuronal activity, thereby increasing serotonin release. Sufentanil is a serotonergic agent that combines with μ receptors to exert analgesic effects. Furthermore, it is a 5-HT1A receptor agonist that can increase the release of serotonin. Mildly inhibiting serotonin reuptake increases synaptic serotonin levels.^[32]^ The patient was regularly taking oxycodone extended-release tablets in the past and had not experienced serotonin syndrome, probably because oxycodone does not inhibit serotonin reuptake but only increases inter-synaptic serotonin via neurotransmitter release or some other unknown mechanisms, resulting in the weaker effect of serotonin syndrome compared with sufentanil. Nevertheless, after serotonin syndrome occurrence, sufentanil and doxepin tablets were discontinued. After adding diazepam, symptoms slightly improved but not significantly, with blood pressure remaining high. Nevertheless, after the administration of nitroglycerin and naloxone, the patient symptoms were significantly relieved. Promptly recognizing and managing patients with serotonin syndrome in time is vital; otherwise, it can lead to serious consequences.

Researchers have reported that when the usage of opioid drugs alone is ineffective, the adverse reactions are intolerable, or patients experience significant anxiety, the combination of sedatives can be considered to increase the analgesic effect of opioid drugs, which can decrease the dosage of opioid drugs and alleviate adverse reactions.^[33]^ Dexmedetomidine is an α2 adrenergic receptor agonist, which exerts sedative, analgesic, hypnotic, anti-anxiety, and inhibitory effects on the activity of the sympathetic nervous system. Furthermore, it can alleviate nausea, vomiting, and hyperalgesia induced by opioids. Studies have also revealed that low-dose dexmedetomidine (0.11–0.15 µg/kg.h) combined with opioids exerts a better analgesic effect and significantly improves the quality of life of patients^.[8–11]^ Moreover, studies have revealed that α2-receptor agonists play a crucial role in neuropathic pain because they can alleviate pain and promote homeostasis. Furthermore, dexmedetomidine can inhibit the release of norepinephrine and serotonin.^[34–36]^ Therefore, dexmedetomidine exerts a therapeutic and preventive effect on serotonin syndrome.^[12,13]^ In the present case, the patient had a combination of neuropathic pain, anxiety, and a history of serotonin syndrome. Therefore, the subsequent selection of dexmedetomidine and sufentanil for analgesia is of great significance.

## 5. Conclusion

Most patients with bone metastasis-associated cancer pain experience mixed pain, accompanied by both nociceptive and neuropathic pain and depression. Therefore, using multimodal combination therapy, including drugs with multiple mechanisms of action, minimally invasive interventions, and psychotherapy, is essential for controlling refractory cancer pain. While paying attention to common adverse reactions in pain treatment, including constipation, nausea, vomiting, lethargy, itching, dizziness, urinary retention, delirium, cognitive impairment, and respiratory depression, we should still pay attention to rare adverse reactions, including the occurrence of serotonin syndrome. In particular, when combined with different analgesic drugs, the incidence of rare adverse reactions increases. At present, although various therapies have been developed for this condition, complete relief from CIBP in patients with cancer remains unachieved. Therefore, investigating the mechanism underlying CIBP is essential to develop efficient analgesic drugs. Notably, CIBP has some limitations, which should be considered in future studies, including evaluating the differences between genotype and phenotype. Because pain, particularly in patients with cancer-associated pain, involves psychological, cognitive, and spiritual domains, a multidimensional approach must be utilized to manage pain in these patients. Studies on psychological action may have an important effect to further improve the quality of life of these patients.

## Acknowledgments

The authors appreciate the patient consent to present this case.

## Author contributions

**Conceptualization:** Dan Wang.

**Formal analysis:** Dan Wang, Jing Li.

**Investigation:** Ting Jiang.

**Methodology:** Lingli Zheng, Yan Li.

**Project administration:** Chaomin Liu, Jing Li.

**Visualization:** Xiaomei Fan.

**Writing – original draft:** Dan Wang.

**Writing – review & editing:** Jing Li.
